# Primary Basal Cell Carcinoma Masquerading as Metastatic Basal Cell Carcinoma in the Left Axilla

**DOI:** 10.7759/cureus.44732

**Published:** 2023-09-05

**Authors:** Brianne Haskell Hanisch, Marcus L Frohm, Tamara Poling

**Affiliations:** 1 Medical School, The University of South Dakota Sanford School of Medicine, Sioux Falls, USA; 2 Mohs Surgery, Sanford University of South Dakota Medical Center, Sioux Falls, USA; 3 General Dermatology, Rapid City Medical Center, Rapid City, USA

**Keywords:** basal cell carcinoma histopathology, basal cell carcinoma diagnosis, infiltrative disease, positive sentinel lymph node biopsy, alnd: - axillary lymph node dissection, dermato-oncology, onco dermatology, hedgehog pathway, metastatic basal cell carcinoma, basal cell neoplasms

## Abstract

Basal cell carcinoma (BCC) is considered the most common malignancy in Caucasians. Despite its high prevalence, BCC has extremely low rates of metastasis.

The patient was a 71-year-old male with extensive BCC and squamous cell carcinoma (SCC) skin cancer history who had an extensive, palpable left axillary mass concerning enlarged lymph nodes. No skin lesions were visualized. A lymph node biopsy revealed a sclerosing/infiltrative BCC with perineural invasion extending to the inked margins of the excision and one of four lymph nodes involved by BCC through direct extension. Sectioning revealed a 3.0 x 2.8 x 2.9 cm, ill-defined, fibrotic pink-white mass within the soft tissue. Two tan to pink possible lymph nodes were also identified within the soft tissue, measuring 0.7cm and 0.9cm. There was no definite direct invasion noted, making metastatic BCC suspicious. A left axillary lymph node dissection was performed. In short, he had a nonmobile tumor that showed evidence of invasion of the adjacent pectoralis muscle near the chest wall, abutting the left axillary vein, with extension. In July 2022, approximately one year after diagnosis, the patient received a PET scan and had no remote sites of disease. Every follow-up PET scan since has shown stable disease, most recently in May 2023. The patient continues dermatology follow-ups every three months for clinical surveillance.

This case is unique because metastatic disease was never confirmed, though it is still a possibility. The affected lymph nodes were in the regional basin, where the patient had had extensive skin cancers in the past. Their involvement could have been secondary to direct invasion, though this could not be confirmed histologically, making the definitive characterization of this particular tumor difficult. As the PET CT scans have remained stable without evidence of distant disease, we favor that this is a recurrent primary tumor with direct extension to the underlying pectoralis and axillary lymph nodes. As common as BCCs are, this case highlights the importance of diligent treatment and follow-up to avoid the potential for tumor-related morbidity and, rarely, mortality.

## Introduction

Basal cell carcinoma (BCC) is the most common skin cancer in humans, arising (and receiving its name) from the basal layer of the epidermis and its appendages. In 2019, new diagnoses of BCC reached over 1.7 million in the United States alone, making it the most common skin cancer in the nation [1]. Basal cell carcinoma presents as a shiny, pink papule or nodule with surface telangiectasia and rolled borders. A biopsy should be performed in all patients with suspected BCC to confirm the diagnosis and determine the histologic subtype. Treatment is surgical, most commonly via wide excision or Mohs surgery. There are a select few, more superficial BCCs that are able to be treated medically [2]. Radiation is typically reserved for inoperable tumors or non-surgical candidates.

Environmental risk factors for BCC are well known. Acute, intermittent ultraviolet (UV) exposure during childhood, adolescence, and young adulthood carries an increased BCC risk [3]. Higher cumulative UV exposure and a higher intensity of UV are independent risk factors. Furthermore, skin type, or the ability of the skin to tan or burn, contributes to the development of disease [2,3]. The best predictor of BCC is a prior history of non-melanoma skin cancer (NMSC), with patients having a 10 times greater likelihood of developing a second BCC if they have a prior history compared to those without NMSC history [2].

Basal cell carcinoma incidence increases significantly with age. The median age at the time of diagnosis is 68 years [2]. However, the most notable increase has been observed in young women in both Europe and the United States [4]. While metastatic potential is extremely low, BCC is locally invasive and destructive, with significant morbidity if left untreated. Thankfully, mortality concerns are also uncommon, primarily affecting immunocompromised patients. Metastatic BCC (mBCC) can affect any patient with BCC and happens more often in tumors with aggressive histopathologic morphology (morpheaform, metatypical, basosquamous, and infiltrating) [2]. For the rare BCC that does metastasize, the most common sites of metastatic disease are the regional lymph nodes, lungs, bone, and skin [5].

Hedgehog pathway inhibitors are a relatively recent advancement in the treatment of metastatic and inoperable BCC. In 2012, the FDA approved vismodegib, followed by the closely related sonidegib in 2015 [2]. These drugs are useful for metastatic tumors, inoperable tumors, poor surgical candidates, and palliative care purposes; however, the side effects of both drugs can lead to discontinuation in up to 55% of patients. One report highlighted the case of a 55-year-old Caucasian male with mBCC to the axillary lymph node who suffered from alopecia, muscle spasms, and mild dysgeusia while on vismodegib [6]. Other common side effects include fatigue, nausea, diarrhea, and weight loss, to name a few [2].

This case report presents a possible mBCC to the axillary lymph nodes along with diagnosis, treatment, and surveillance considerations.

## Case presentation

**Figure 1:**
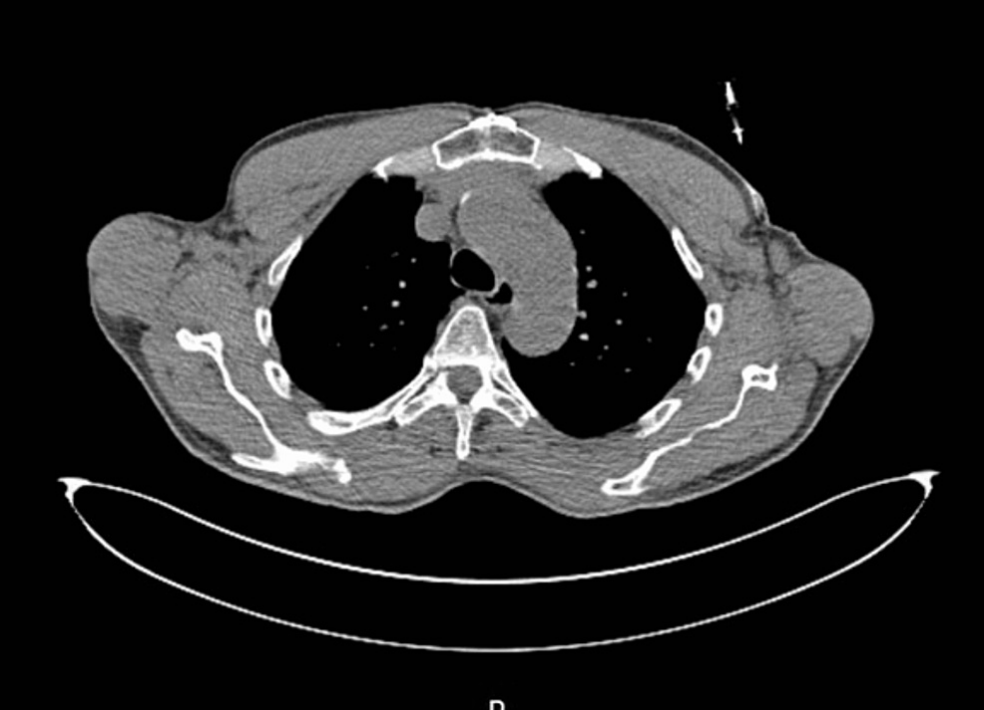
CT-guided lymph node biopsy A transverse section of the upper chest shows needle positioning for biopsy in the left axillary node in May 2021.

A 71-year-old Caucasian, blue-eyed male with a history of numerous NMSCs presented to urgent care with left shoulder pain in May 2021. During that visit, the patient had an extensive, palpable left axillary mass concerning lymphadenopathy. No skin lesions were noted in the medical record. He was referred to general surgery, where a CT-guided core biopsy revealed sclerosing/infiltrative BCC with perineural invasion and one of the four lymph nodes involved by BCC through direct extension (Figure 1).

**Figure 2:**
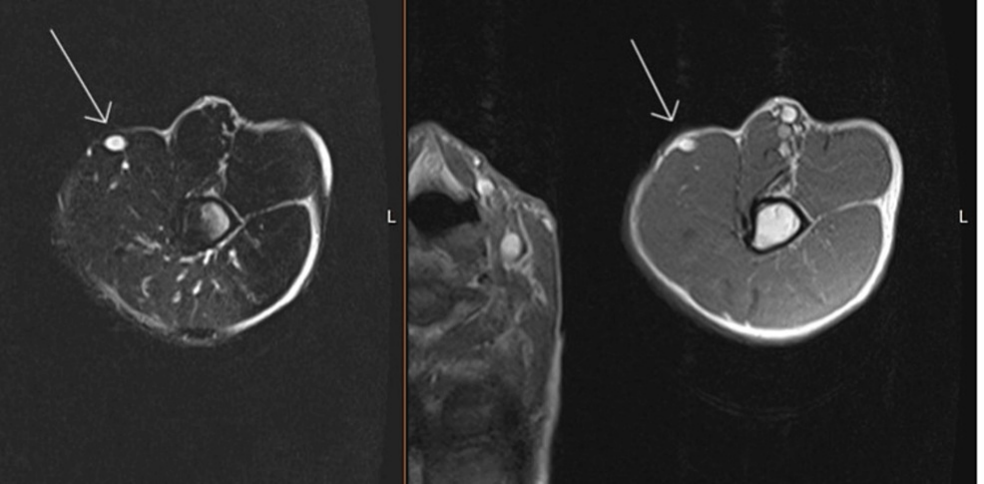
Soft tissue MRI of the chest The left side of the image shows a T2-weighted transverse section; the right side is the T1-weighted image taken in May 2021. A hyperintense lymph node is exhibited in both images in the 11 o'clock position.

**Figure 3:**
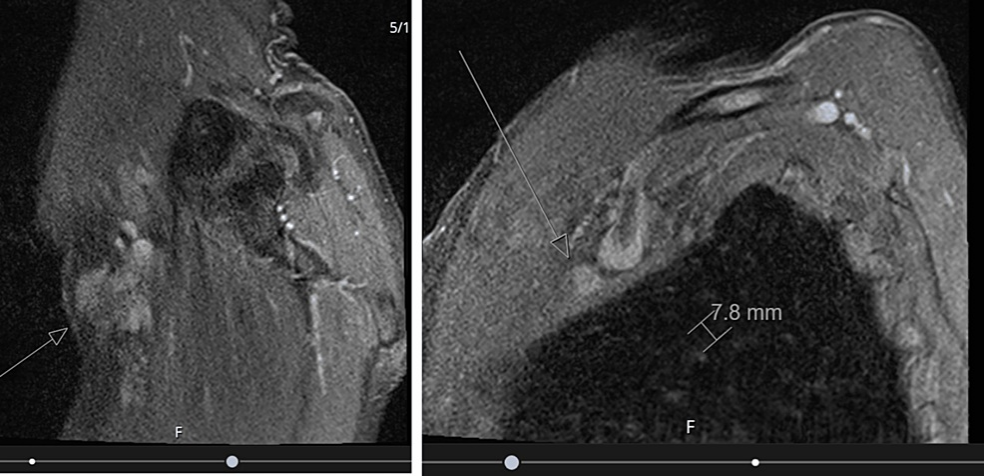
Soft tissue MRI of the chest Highlights a different section of the same image in Figure 2. Arrows indicate mass abutting the anterior head of the deltoid muscle, located immediately deep to the neurovascular structures without definite direct invasion.

Soft tissue MRI revealed a 3.0 x 2.8 x 2.9 cm ill-defined, fibrotic pink-white mass within the soft tissue (Figures 2-3).

**Figure 4:**
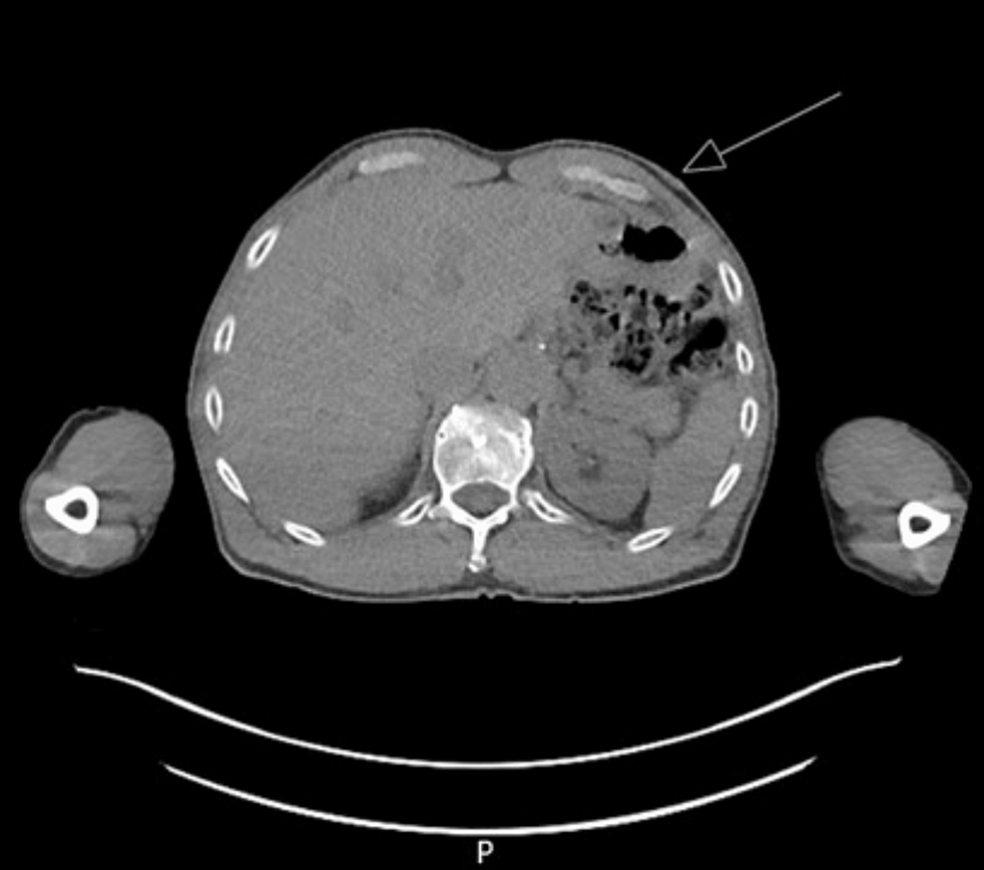
A CT PET from the skull base to mid-thigh, May 2021 Moderate hypermetabolic activity is associated with the high left axillary biopsy-proven malignancy. A second, more medially located hypermetabolic lymph node in the left upper chest, subpectoral region, is suspicious for metastasis as well. There is no definite linear extension along the neurovascular bundle.

Two possible lymph nodes were also identified within the soft tissue measuring 0.7cm and 0.9cm. There was no definite direct cutaneous invasion noted raising the possibility of mBCC (Figure 4).

A left axillary lymph node dissection was performed in June 2021. The mass appeared to be attached to a portion of the pectoralis along the lateral edge, and a margin of muscle was taken. The mass closely approximated the axillary vein, though this was surgically spared. The subscapular vein was directly involved, as were multiple nerves in the axillary vault. The tumor involved pectoralis minor, and dissection continued to levels two and three to free the tumor. Several lymph nodes appeared to have metastatic disease.

In short, the patient had a large, fixed tumor that showed evidence of invasion of the adjacent pectoralis muscle near the chest wall, abutting the left axillary vein, with extension to the lymph nodes. Given the high risk of recurrence, adjuvant radiation therapy was discussed in addition to referral to oncology. Although radiation was offered and strongly encouraged, the patient ultimately elected to defer treatment. He continued to follow up with dermatology every three months for clinical surveillance.

Approximately one year after diagnosis, in July 2022, a PET CT revealed an increase in the size and F-fluorodeoxyglucose activity of several left axillary, subpectoral, and supraclavicular lymph nodes. No distant disease was detected. Oncology recommended vismodegib. The patient started treatment in September 2022. A follow-up PET CT in February 2023 revealed stable disease. In March 2023, the patient complained of fatigue, nausea, and dysgeusia. The most difficult side effect to manage for him was the altered taste of food. He remained on vismodegib, and his most recent scans in May 2023 again revealed stable disease.

## Discussion

Basal cell carcinoma is the most common malignancy in Caucasians, therefore placing a large burden on healthcare systems [4]. It constitutes 80% of all NMSCs, but, despite its high prevalence, rates of metastases are exceptionally low, varying from 0.0028% to 0.55% [7]. Our current understanding of mBCC is derived from two major literature reviews. The first review reported 170 cases from 1894 to 1980, and a subsequent review described 194 cases between 1981 and 2011 [7]. True mBCC has a poor prognosis and a mean survival rate of three years in cases with regional metastases to the lymphatics [6].

Although rare, there are certain tumor characteristics that should raise clinical suspicion for mBCC. Our patient's individual risk factors include fair skin, multiple prior BCCs on the left chest and left pectoral region and high-risk histology (infiltrative) from several of the previous sites. In our patient’s case, metastatic disease was never confirmed, though it is still a possibility. The affected lymph nodes were in the regional basin, where the patient had had extensive skin cancers in the past. Their involvement could have been secondary to direct invasion, though this could not be confirmed histologically, making the definitive characterization of this particular tumor difficult. As the PET CT scans have remained stable without evidence of distant disease, we favor that this is a recurrent primary tumor with direct extension to the underlying pectoralis and axillary lymph nodes. In any case, first-line therapy continues to be surgical excision. In the rare instances where surgical interventions fail, radiation and systemic therapy with hedgehog pathway inhibitors are appropriate to consider while weighing the patient-specific risk-benefit ratios. By presenting this patient's case, we hope to draw awareness to the vitalness of early clinical detection of NMSCs and showcase a tumor that initially raised suspicion for mBCC but is now favored as BCC with direct extension.

## Conclusions

Our patient had a long history of NMSC at various locations with aggressive histology and required close clinical surveillance. Without an obvious skin lesion on presentation and the inability of histology to confirm the tumor by direct extension, this patient was worked up and followed as if they had mBCC. Diligent treatment and follow-up to avoid the potential for marked tumor-related and treatment-related morbidity and, rarely, mortality is essential. We present this case to reinforce the utmost importance of this surveillance and prevention of NMSC as well as showcase how deceiving metastatic disease may be. This case report may serve as a guide for future cases and an encouragement for research in the area.
